# Integration sites of the HTLV-1 provirus in host human genome in the IDH patients with monoclonal integration

**DOI:** 10.1186/1742-4690-8-S1-A144

**Published:** 2011-06-06

**Authors:** Juliana M Argolo, Maria de Fatima P de Oliveira, Achilea C Bittencourt, Lourdes Farre

**Affiliations:** 1 1Laboratory of Experimental Pathology, CPQGM, FIOCRUZ, Salvador, Bahia, 40296710, Brazil; 2 2Department of Internal Medicine, HUPES, Federal University of Bahia, Salvador, Bahia, 40296710, Brazil; 3 3Department of Pathology, HUPES, Federal University of Bahia, Salvador, Bahia, 40296710, Brazil

## Background

Infective dermatitis associated with HTLV-1 (IDH) is a recurrent and severe childhood infected form of eczema, that usually begins at 18 months in vertically-infected children. IDH may progress to adult T-cell leukemia/lymphoma (ATL). Abnormal T cells (ably), including flower cells, were found in the peripheral blood smears of 30% (9/31) of IDH patients in Bahia, Brazil. Monoclonal integration of provirus assessed by inverted long PCR (ILPCR) was detected in 3 of these patients (2 with flower cells and one with ably cells but without flower cells in peripheral blood smear). The aim of this study was to identify the integration sites of the HTLV-1 provirus in host human genome in the IDH patients with monoclonal integration

## Materials and methods

The sequences of the PCR products of these 3 patients obtained by ILPCR were mapped on to the human genome using the Basic Local Alignment Search Tool http://www.ncbi.nih.gov/blast.cgi.

## Results

In the two patients with flower cells, integration sites were detected, respectively, in chromosome 1 (1p34.2) and chromosome 3 (3p11.1). In chromosome 1, the provirus was located within PPM1H gene coding region and in chromosome 3, within alpha satellite DNA sequences. The patient with integration in chromosome 1 also presents HAM/TSP. In the IDH patient without flower cells, integration site was identified at chromosome 12 (12q14) and was located near the transcriptional start site of GUCA2B gene.

## Discussion

In IDH patients with monoclonal integration, the provirus integration sites can be associated with transcriptionally active regions of the human host genome.

